# A Review of the Management of Obesity in Primary Care

**DOI:** 10.1111/cob.70040

**Published:** 2025-08-20

**Authors:** H. M. Parretti, S. E. Erskine, K. D. Coulman, R. Mears, K. Clare, K. Williamson, R. Watkins, C. A. Hughes

**Affiliations:** ^1^ Norwich Medical School University of East Anglia Norwich UK; ^2^ Health Economics and Health Policy Bristol and Centre for Academic Primary Care, Population Health Sciences, Bristol Medical School University of Bristol Bristol UK; ^3^ Centre for Academic Primary Care, Bristol Medical School University of Bristol Bristol UK; ^4^ Leeds Beckett University Leeds UK; ^5^ NRS Clinician NHS Lothian Weight Management Team Edinburgh UK; ^6^ Honorary Research Fellow, School of Health & Wellbeing University of Glasgow Glasgow UK; ^7^ Fakenham Medical Practice, Meditrinia House Fakenham UK

**Keywords:** education, general practice, GLP‐1 agonist, obesity, primary care

## Abstract

This review highlights the important role primary care plays in obesity management, using England as an example. It includes a comprehensive summary of current management and referral options for primary care clinicians, a discussion of the most up‐to‐date clinical guidelines for the use of GLP‐1 receptor agonists in England, and the evolving ways in which obesity is identified and defined. Reflections from people living with obesity are considered. Despite the potential of primary care to engage with patients regarding obesity prevention and treatment, several factors have limited this, including low prioritisation by clinicians, workload pressures, regional variations in services, insufficient specialist training and ongoing weight stigma. The introduction of new pharmacotherapies, such as GLP‐1 receptor agonists, offers both an opportunity and a challenge for primary care providers. These treatments could help patients access more effective obesity management strategies via primary care. However, there is concern about non‐specialist clinicians keeping up to date with evolving strategies and understanding how new medications fit into broader care. The current complex referral pathways hinder timely access to appropriate treatment. The need for more straightforward pathways, improved clinician education and a reduction in the stigma associated with obesity is critical for better outcomes. In summary, while primary care could play a pivotal role in addressing obesity, several issues need to be resolved for this potential to be fully realised. Addressing these challenges, via enhancing clinician training, improving referral pathways and ensuring access to new treatments, will be crucial for advancing the care of people living with obesity.

**Abbreviations**
BMI: body mass index
CCGs: clinical commissioning groups
HCPs: healthcare professionals
ICBs: integrated care boards
NES: National Enhanced Service
NHS: National Health Service
NHSE: NHS England
NICE: latest National Institute for Health and Care Excellence
PLwO: on people living with obesity
SWMS: Specialist Weight Management Services
WMS: weight management services

## 1 Introduction

### 1.1 Overview of Obesity and Its Health and Social Impacts

An analysis of worldwide trends in underweight and obesity recently published in the *Lancet* reported that the age‐standardised prevalence of obesity in adults increased between 1990 and 2022 in 188 countries for women and in 187 countries for men [1]. In the United Kingdom, 26% of adults are living with obesity; 3% have severe obesity (BMI ≥ 40 kg/m^2^) [2].

Obesity is associated with multiple co‐morbidities and can reduce quality of life and life expectancy [3]. The estimated annual costs of obesity to the National Health Service (NHS) in England are between £2.47 billion and £5.1 billion [4]. These are predicted to rise to £10 billion by 2050, with wider costs to society estimated to reach £50 billion [5]. Societal impacts are felt through higher levels of worker absenteeism, reduced productivity, increased dependence on disability benefits, early retirement, increased levels of chronic disease and mental health concerns, including those arising from obesity‐related experiences such as weight stigma [6, 7, 8]. Although everyone is at risk of developing obesity, those of lower socioeconomic status and those from ethnic minorities experience a greater burden [9].

For people with severe obesity, the physical consequences of obesity, such as osteoarthritis, lymphoedema and cardiac disease can lead to functional mobility limitations [10]. Alongside the increased occurrence of anxiety and depression [11] and combined with the impact of weight stigma [12], these factors can contribute to poorer psychosocial function, including social isolation [13, 14], potentially feeding back into worsened physical health [15]. In lived experience terms, the psychosocial impact has been described as ‘The body as an impediment to living the desired life, to being oneself and to moving on in one's life’ [16].

### 1.2 Primary Care and Its Potential Role in the Management of Obesity

The World Health Organisation defines primary care as ‘a model of care that supports first‐contact, accessible, continuous, comprehensive and coordinated person‐focused care. It aims to optimize population health and reduce disparities across the population by ensuring that subgroups have equal access to services’ [17]. NHS England (NHSE) also describes primary care services as providing the first point of contact in the healthcare system. In the NHS, primary care conducts the majority of all healthcare contacts (329 million primary care appointments in 2022) [18]. Primary care clinicians often have the benefit of long‐term relationships with their patients and a good understanding of the context of their lives. They are experienced in chronic disease management. These factors make them ideally placed to help manage obesity. Primary care therefore has a vital role with clear opportunities to discuss with and support those with overweight and obesity, signpost to resources and navigate access to specialist services alongside providing ongoing care.

### 1.3 Identification of Obesity

Obesity has traditionally been classified by body mass index (BMI) alone; the flaws of this practice are well‐known, with BMI‐based measures of obesity both under‐and over‐estimating adiposity, offering insufficient insight into overall individual health [19]. To address this concern, the Lancet Diabetes and Endocrinology commissioned a report by 58 experts [20]. The specific aim of the commission was ‘to establish objective criteria for disease diagnosis, aiding clinical decision making and prioritisation of therapeutic interventions and public health strategies’. The commission concluded that excess adiposity should be confirmed by either direct measurement of body fat, where available, or at least one anthropometric criterion (e.g., waist circumference, waist‐to‐hip ratio, or waist‐to‐height ratio) in addition to BMI [20]. This is in alignment with the latest National Institute for Health and Care Excellence (NICE) guidelines, with detailed reference to measuring waist‐to‐height ratio for those with BMI < 35 kg/m^2^ [19]. This new NICE guideline (NG246) places a stronger emphasis on personalised care, ensuring that weight management strategies are tailored to an individual's specific needs, preferences and circumstances, starting with more accurate identification of those at risk (including a useful visual summary of general principles of care) [19]. Primary care healthcare professionals (HCPs) are in an ideal position to better identify at‐risk individuals via implementation of these recommendations.

### 1.4 Weight Stigma

There is increasing recognition of the impact of weight stigma on people living with obesity (PLwO) including experiences within healthcare settings and from interactions with HCPs [21]. Evidence shows that experiences of weight stigma in healthcare can lead to avoidance of future healthcare, lower trust in HCPs, reduced quality of care and consequently lead to health disparities. A World Obesity Federation survey of 50 countries (2021) concluded that weight stigma in the healthcare system was a factor in the lack of services for PLwO in most countries [22]. It is important that the impact of weight stigma on access to care is recognised and that awareness of weight stigma is increased amongst HCPs to improve future care provision and patient experiences.

In summary, the complexities of the consultation, preconceptions of both patients and clinicians around discussing obesity, time pressures in primary care and weight stigma are important considerations in obesity management within primary care.

Through a narrative review of published literature and expert knowledge of the author team, this paper aims to discuss the management of obesity (BMI) (≥ 30 kg/m^2^) within primary care, using the healthcare system in England as an example. Although focused on England, there will likely be correlations with the management of obesity in other healthcare systems of interest to an international audience. The authors include a clinical nurse specialist in obesity, a person with lived experience, GPs with a specialist interest in obesity and a bariatric dietitian. All authors have experience and expertise in obesity research within the context of primary care. Additional patient perspectives were included via two people from Obesity UK (a national patient organisation for people living with obesity in the United Kingdom) reading the manuscript and sharing their comments and responses to the content from their perspectives in a focus group with HP and SE. This was not formal qualitative research, but patient and public involvement in preparing the manuscript.

The next section will discuss the key management options via primary care in England; other countries will likely have comparable services available. HCPs in primary care also have a role in the prevention of obesity and/or in the prevention of disease and other sequalae of obesity [23]. However, the prevention of obesity is outside the scope of this review and warrants its own review.

## 2 Management Options for Overweight and Obesity in Primary Care

### 2.1 Tiered System

Historically, the weight management services in England have been commissioned and funded based on a four‐tiered system, see Box 1 [24, 25]. Funding was organised through clinical commissioning groups (CCGs) which were clinically led NHS bodies responsible for the planning and commissioning of health care services for their local area. These have been replaced by integrated care boards (ICBs) which include a broader range of stakeholders such as social care services.

BOX 1 **The tiered system for weight management services in England [24, 25].**

- *Tier 1*—funded by public health as a universal intervention aimed at providing healthy lifestyle principles, such as healthy dietary intake and increasing physical activity.
- *Tier 2*—weight management services incorporating lifestyle changes and behavioural change interventions, usually delivered as a group intervention over 12 weeks and often provided by accredited commercial providers. Tier 2 was commissioned by public health, or in some areas by clinical commissioning groups (CCGs).
- *Tier 3*—specialist weight management services (SWMS) with a defined core team including specialist physicians and a multidisciplinary team (MDT) including specialist doctors, dietitians, nurses, psychologists and physiotherapists that could be delivered in either primary or secondary care. Tier 3 services provided specialist diets such as low energy diets, pharmacotherapy, behavioural and psychological interventions and preparation for referral for bariatric surgery. Tier 3 was commissioned by CCGs.
- *Tier 4*—a bariatric surgery unit with full MDT support to provide assessment for surgery, surgical interventions and pre‐ and post‐surgery care. Tier 4 has been centrally funded initially and then transferred to local CCGs. Post bariatric surgery follow‐up is commissioned in the surgical centre for 2 years and then it is recommended people are discharged to a shared care annual follow up in primary care.

Within these tiers, there are many different management strategies available to primary care HCPs:

1. Opportunistic Brief Interventions
  To date, opportunistic brief interventions around obesity in primary care have mainly been recommended via the Making Every Contact Count (MECC) initiative from Public Health England and National Health Service England (NHSE) [26]. MECC has been used to improve health and wellbeing by targeting behaviours such as alcohol use and smoking, as well as weight management. The key aim of the initiative was that all HCPs should be able to deliver a brief intervention to support healthier behaviours. In 2016, the BWeL trial was the first randomised controlled trial to investigate whether such a brief intervention delivered by GPs to offer weight management support was effective. In this trial, participants were offered referral to a commercial weight management programme for 12 weeks during a consultation with their General Practitioner (GP) [27]. One thousand eight hundred and eighty‐two participants were recruited, and the trial found that at 1 year, the intervention group lost on average 2.43 kg compared with 1.04 kg in the comparator group (advice only). In addition, < 1% of patients thought a brief intervention to offer referral to a weight management service was inappropriate and unhelpful [27].
2. Community‐Based Group Interventions
  Patients with overweight or obesity can be referred to a community‐based weight management intervention, depending on local eligibility criteria and service availability. These are usually group‐based and include behavioural support on healthy eating and physical activity. The provision of these services under the NHS is variable across England. Usually, NHS provision is for 12 weeks, with patients being able to continue with the programme beyond this time if they are able to pay privately to do so. There is evidence from randomised controlled studies on the effectiveness of these programmes; the Lighten Up trial compared multiple different commercial providers of community group‐based weight management programmes with a physical activity programme, GP or pharmacy‐led weight management. All programmes achieved significant weight loss (between 1.37 kg [GP led] to 4.43 kg [Weight Watchers]), and those except GP‐led and pharmacy‐led resulted in significant weight loss at 1 year. The commercial programmes achieved significantly greater weight loss than the primary care programmes (mean difference 2.3 (1.3–3.4) kg) and were less expensive. Self‐reported physical activity was measured in this study and was increased at the end of the programme, although at 1 year there were no significant differences with the comparator group. Only the pharmacy group reported statistically significantly more activity in the unadjusted analysis. The authors concluded that commercially provided weight management services are more effective and cheaper than primary care based services [28].
3. National Programmes
  There has been an increase in the provision of national programmes related to obesity and weight management, including primarily digital interventions. Such interventions target people with obesity and specific co‐morbidities, namely Type 2 diabetes and hypertension.
  ○ *NHS Digital Weight Management programme* which is available for adults (aged ≥ 18 years) with obesity (BMI ≥ 30 kg/m^2^, lowered to 27.5 for those from Black, Asian and ethnic minority backgrounds), and diabetes and/or hypertension. The 12‐week online programme requires access to a smartphone or computer (provider is dependent on the geographical area within which the person lives) [29]. It has shown promising results [30].
  ○ *NHS Healthier You programme* (previously Diabetes Prevention Programme) is available for adults at high risk of developing Type 2 diabetes (HbA1c ≥ 42–47.9, or fasting plasma glucose results between 5.5 and 6.9 mmols/l or history of gestational diabetes). While the focus of the nine‐month programme is on reducing the risk of Type 2 diabetes rather than weight alone, due to the close relationship between weight and Type 2 diabetes, much of the input is relevant to weight management. Patients can usually choose between in‐person or digital delivery of the programme. Evaluations of this programme have been conducted and there is evidence supporting its efficacy in both weight loss and reducing population incidence of Type 2 diabetes [31, 32].
  ○ *NHS Type 2 Diabetes Path to Remission programme* (low energy diets for people with Type 2 diabetes) [33]. This programme is available for those 18–65 years, who have a diagnosis of Type 2 diabetes within *the* last 6 years and have a BMI over 27 kg/m^2^ (where individuals are from White ethnic groups) or over 25 kg/m^2^ (where individuals are from Black, Asian and other ethnic groups). This pilot programme was developed based on the results of two key randomised controlled trials DIRECT and DROPLET, which showed the effectiveness of low energy diets in primary care (as total diet replacement) for the remission of Type 2 diabetes and weight change (adjusted mean difference in weight in DROPLET was −7.2 kg (95% confidence interval −9.4 to −4.9 kg)), while in DIRECT a third with early Type 2 diabetes achieved remission [34, 35]. These trials also showed that a total diet replacement was feasible and safe to be delivered in primary care [36].

In 2021, a National Enhanced Service (NES) was launched to encourage GPs to refer patients to weight management services, which continues to be in place [37]. In this NES, GPs are given a financial incentive per patient referred to a weight management scheme. A recent evaluation found no strong evidence that the NES affected how clinicians addressed weight management or related behavioural risk factors within annual review consultations for PLwO [38].

4 Specialist Weight Management Services (SWMS)
  SWMS aim to provide a personalised multidisciplinary team (MDT) approach to the management of obesity. In England, adults with a BMI ≥ 35 kg/m^2^ with weight‐related co‐morbidities or BMI ≥ 40 kg/m^2^ are eligible for referral to these services according to current NICE guidelines [19]. However, provision across the country is variable [38].

Currently, primary care clinicians can refer patients to all tiers; however, often patients are required to complete one tier before they can be referred to another. These include national programmes such as the NHS Digital Weight Management Programme (tier 2 level), the NHS Healthier You programme and the NHS Type 2 Diabetes Path to Remission programme, all discussed above. In England, the National Institute of Health and Care Excellence (NICE) provides guidance to clinicians regarding the identification, assessment and management of people living with overweight or obesity. It is hoped that the recent NICE update in which a SWMS is defined more flexibly than the original tier 3/4 (see Box 2) will help with access to specialist MDT input [19].

SWMS aim to provide specialist medical, dietetic, psychological and physical activity input. Some services also have access to specialist pharmacists. Generally, in England, SWMS are based in hospitals, but there have also been some based in the community [39]. In addition to conducting a detailed initial assessment of the patient's needs related to their obesity and providing behavioural support, SWMS aim to optimise health and wellbeing, including providing specialist dietetic and/or psychological input. They are also the gateway to both the new pharmacotherapy treatments now available for obesity and/or to bariatric surgery. At present, most patients are provided with 12 months of support and, at the end of this 12 months period, may be referred for consideration for bariatric surgery or discharged back to their GP. SWMS also provide the first 2 years follow‐up care post‐bariatric surgery.

Primary care has a role in the long‐term follow up of patients who have had bariatric surgery (in addition to referring into the service initially). Despite its well‐recognised health benefits, without adequate follow‐up, bariatric surgery has long‐term risks including nutritional deficiencies and weight regain [40, 41, 42, 43]. There is also evidence from cohort studies and systematic reviews that lack of follow‐up care has a negative impact on patient outcomes [44, 45, 46]. In the United Kingdom, NICE guidance recommends that bariatric surgery patients stay under specialist surgical care for the first 2 years post‐surgery and are then discharged to primary care for annual reviews under a shared care model with a bariatric specialist [19].

BOX 2 **National Institute for Health and Care Excellence definition of SWMS [19].**

‘A specialist primary, community or secondary care based multidisciplinary team offering a combination of surgical, dietetic, pharmacological and psychological obesity management interventions, including but not limited to tier 3 and tier 4 services’ NICE generic principles of care.

### 2.2 Pharmacotherapy

With the advent of the new pharmacotherapy for obesity, SWMS now also have a role in prescribing and monitoring these medications. Until recently, the options for pharmacotherapy for PLwO in England were more limited than in other countries, and the only medication which GPs could prescribe was orlistat. However, the GLP‐1 receptor agonists, liraglutide, semaglutide and tirzepatide have now all been approved for use within the NHS [47, 48]. The latest NICE guidelines [19] recommend that semaglutide and liraglutide are prescribed via SWMS, while tirzepatide can be prescribed from primary care (for adults with an initial BMI of at least 35 kg/m^2^ and at least one weight‐related comorbidity). After the introduction of semaglutide, the UK government announced a £40 million pilot to test provision of GLP‐1 receptor agonists outside of hospital settings [49]. Since tirzepatide will also be prescribable from primary care, there will be a large population of potentially eligible patients, and there are significant concerns from commissioners and HCPs about availability of services, clinical capacity, inequity of access and budget impact, leading to NHS England submitting a funding variation request, on behalf of NHS providers and ICBs, to extend the time needed to comply with NICE recommendations [48]. Under this funding variation, the full implementation period will be 12 years, with PLwO prioritised by clinical need in terms of BMI and co‐morbidities [48]. In addition, NICE has recently completed an early value assessment on digital interventions to support treatment with these medications [50]. Several digital companies were recommended to be used to prescribe and monitor medication and to help deliver multidisciplinary weight‐management services while more information is gathered [50]. Given the current evidence for the effectiveness of these new GLP‐1 receptor agonists, it will be key that access to these medications is increased to improve the health of PLwO in the United Kingdom [51].

### 2.3 Weight Loss Maintenance

Even after successful weight loss, many people struggle to maintain their weight. Primary care also has an important role to support people with weight maintenance and consider referral for further interventions and/or signpost to resources, where indicated. For example, those on the European Association for the Study of Obesity webpages (e.g., https://cdn.easo.org/wp‐content/uploads/2020/06/25172155/bariatric‐surgery‐tips_v3.pdf). Chronic disease management consultations often include a weight check and are a prime opportunity to review the weight trajectory of a patient and, if pertinent, offer help. It is also important to affirm weight maintenance.

There are powerful biological, psychological, environmental and social factors that continue to drive weight gain [52]. In a meta‐analysis of 29 long‐term weight loss studies, over 50% of weight lost was regained within 2 years of completing the intervention [53]. There is some evidence that third wave cognitive behavioural therapy (CBT) interventions may help; acceptance and commitment therapy (ACT) showed the most consistent results in a recent review, demonstrating effectiveness beyond 18 months [54]. Weight regain is a very significant issue which must be considered both by those designing weight loss programmes and by those involved in ongoing follow up, which will always involve primary care. While currently commissioned weight management services vary in content and emphasis on weight maintenance and are of limited duration with minimal long‐term support, the recently updated NICE guidance has recommended that providers discuss sources of ongoing, post‐intervention support with patients [19, 55]. Physical activity has repeatedly been shown to be helpful for weight maintenance [56] and there is an opportunity for HCPs in primary care to discuss this and signpost people to appropriate resources, such as Moving Medicine (https://movingmedicine.ac.uk/) as well as initiatives such as Park Run.

### 2.4 Barriers to Accessing Weight Management Services (WMS) From Primary Care

Access to WMS remains a significant problem in England. A recent study by Coulman et al. found that access is poor, with no indications of improvement over the past 10 years [57]. This study used routine GP data and found that only 3% of those with a recorded diagnosis of overweight/obesity in England between 2007 and 2020 had a referral for WMS recorded [2, 57]. This pattern has been widely reported; Booth et al. found that approximately 60% of people with a BMI ≥ 40 kg/m^2^ did not have any WMS intervention recorded during the study period and only around 17% of people with a BMI ≥ 40 kg/m^2^ received a WMS referral [58].

Given widespread under‐recording of weight/BMI in primary care clinical records, the issue may be even greater; those without weight/BMI recorded are unlikely to have accessed any WMS support since most referrals are contingent on documented BMI [57]. Under‐recording may be due to different factors, including lack of time in consultations, inadequate training, or low confidence amongst HCPs, a belief that primary care is not an appropriate setting for managing obesity, or a lack of access to appropriate referral programmes. These may in turn influence the offer of WMS referrals [59, 60, 61]. Despite national guidance with respect to the availability of WMS and bariatric surgery, there are known regional inequalities with variations in commissioning and irregular provision of WMS and NHS bariatric centres across the country [62]. Rural–urban location and socio‐economic variation are also likely to contribute to inequalities in access [63].

Findings from the ACTION‐IO UK study (an online survey of 1500 people living with obesity and 306 HCPs with experience of consulting with PLwO published in 2021) suggested that PLwO were not discussing their weight with HCPs until they had lived with overweight/obesity for many years (mean 9 years) [61]. HCPs reported short consultation times and other medical problems to address as barriers to discussion, thus tended not to raise weight until co‐morbidities developed [61]. Many HCPs judged that PLwO were not interested or motivated to lose weight before even offering them a chance to discuss it [61]. This may reflect the widespread obesity stigma which has been identified in both HCPs and the UK population [64, 65]. However, responses from PLwO in the ACTION‐IO UK study suggested that they would welcome a discussion and very few (4%) were offended; a similar finding to other studies [61, 62]. Weight‐related conversations evoked complex feelings for PLwO, needing sensitivity and respectful communication. Previous insensitive discussions can lead to apprehension for PLwO about ever discussing weight, plus negativity associated with terminology such as ‘obese’ [66]. In a systematic review of clinical encounters about obesity, clinicians' advice was seen as generalised and unhelpful [67]; linking weight to a specific condition left participants feeling that more serious underlying diagnoses were potentially overlooked, with the concern that every issue they presented would be attributed solely to their weight [68]. These factors led to mixed feelings in relation to discussing weight. Participants did respond more positively to offers of support for weight loss and active monitoring of weight [69]. The physical consultation room was additionally sometimes a barrier, for example, with uncomfortable furniture for PLwO [61, 67]. Perceptions about quality of care have been found to be significantly lower for people who self‐identify as living with overweight or obesity, with a study of health experiences via a Patient Experience Platform (PEP) in the NHS, finding lower perceptions of care quality compared with those not living with overweight or obesity, across parameters such as speed of accessing treatment, effectiveness and emotional support [68].

A lack of confidence within primary care may also be a limiting factor in accessing WMS; one study found that primary care practitioners were prejudiced against bariatric surgery, with almost half not making any referrals despite stating that 30% of their patients were living with obesity. They appeared to lack knowledge regarding the risks of surgery and did not feel confident in managing patients after surgery [69]. A recent systematic review found similar barriers with lack of knowledge amongst both HCPs and patients [70]. One proposed factor is some GPs feeling that obesity is not a medical problem per se, and is instead the patient's problem, even if the GP accepts that patients may want help from them [59, 70]. A recent qualitative study suggested a diverse range of potential factors affecting clinical reasoning amongst primary care HCPs when seeing PLwO [71]. These included perceived differences in cultural understanding, clothing choices masking body size and distribution of weight suggesting potential co‐morbidities [71]. HCPs may wish to help or support PLwO, but acknowledge an inadequate understanding of obesity care. They may have concerns about the time required for these discussions and worries about raising a sensitive topic [72, 73]. Others have reported feeling that existing treatment and management options are ineffective and they do not want to damage the relationship with their patient by suggesting them [59]. Although the scope of options and understanding of management of obesity has evolved rapidly, this potentially leaves an even bigger gap between best practice and clinicians' knowledge of management options and their ability to discuss them confidently. Similar wide‐ranging barriers have been found in other countries, for example, the USA, New Zealand, Germany and Sweden. Patients have also been found to tend to agree with these barriers [73, 74, 75, 76, 77].

Even when educational interventions have been provided to primary care HCPs, there is limited evidence of their efficacy; such interventions appear neither to have led to improved rates of referral [78], nor to have improved weight loss [79]. Goodfellow et al. found no difference in referral rates between groups when comparing general practices in England randomised to receive or not receive training in implementing weight management guidelines [78]. Moore et al. found no difference in weight between patients in intervention and control groups at 3, 12 or 18 months when comparing groups where one group of practitioners had received training [79].

### 2.5 Holistic Care

HCPs in primary care should adopt a holistic approach to obesity management [19]. For example, this could include:

- support with depression and anxiety through referral to local talking therapy services or community mental health teams,
- help with stress management through talking therapies, and/or signposting to self‐help options such as mindfulness Apps,
- screening for eating disorders or disordered eating and referring to eating disorders services as available locally,
- screening for medical conditions related to obesity, such as metabolic dysfunction‐associated steatotic liver disease (MASLD) and obstructive sleep apnoea and referring for further investigation if appropriate,
- social prescribing, including for those experiencing isolation, loneliness or social anxiety,
- medications review for drugs that may be contributing to weight gain,
- exercise on referral schemes.

### 2.6 Social Care Considerations

In England, the policy context, performance and function of primary care is increasingly integrated with that of social care. The bi‐directional relationship between excess weight and disability [80, 81], exacerbated by the functional physical limitations and psychological comorbidities of anxiety and depression associated with severe obesity [3], can render people housebound. Such people often experience the highest degree of excess weight with BMI > 50 kg/m^2^, complex multimorbidity, poor quality of life and reduced function [13, 14]. This leads to increased demand on both home care and care home providers, evident in current UK and international studies [82, 83]. Lack of data means that quantifying the impact of excess weight on social care is difficult, but Gousia et al. found that people with BMI ≥ 45 kg/m^2^ are > 5 times more likely to use formal care than those with BMI 18.5–24.9 kg/m^2^ [82], with recent evidence of home care package costs of > £36 000 per annum [84]. Alongside social care, primary care provides the majority of care for housebound people through home visits. The potential for significant weight loss resulting from new GLP‐1 medications combined with remote or hybrid treatment options could bring huge improvement to housebound people's function, thereby improving quality of life, while potentially reducing both health and social care costs. Alternatively, failure to consider the specific needs of housebound people risks exacerbating current health inequalities, escalating social care costs and increasing demand on primary health and social care services. For housebound PLwO, engagement with weight management services (WMS) appears low [84]. Primary care has a key role to play, whether through improving referral to WMS or directly improving management of obesity for housebound PLwO.

### 2.7 Childhood Obesity

Supporting children and their families to reach a healthy weight, through primary care, is challenging. The programme in England responsible for screening the weight status of children, the National Child Weight Measurement Programme (NCMP), does not routinely feedback results to primary care clinicians [85], hindering the potential for weight‐related discussions in primary care [31]. Documentation of a child's weight status in primary care health records is poor, with only 10.5% and 26% of 5‐ and 11‐year‐olds respectively having at least one GP BMI record [86]. This is concerning as around half of parents underestimate their child's overweight/obesity status [87].

In England, options for onward referral from primary care to tier 2 or tier 3 weight management services for children are dependent on local commissioning decisions, leading to inequalities in service provision [88]. A recent survey found that only 23% of acute NHS trusts offered a weight management service for children living with obesity, giving an indication of the limited tier 3 service availability [88]. However, the new Complications of Excess Weight (CEW clinics) are beginning to address inequities in service provision through a national coordinated approach to specialist weight management care [89].

## 3 Discussion—The Future of Weight Management and Challenges for Primary Care

The wide variety of weight management programmes and services available and the complexity of their provision, together with the geographical variation of available services (and waiting times), currently make timely and appropriate referral according to an individual's needs very challenging to achieve. This is illustrated by the low numbers of PLwO who successfully access them [57]. There are similar issues internationally; a report on 50 countries found a lack of adequate services in many, especially in lower‐income countries and in rural areas. Lack of treatment was attributed to absent care pathways from primary care to secondary services; absent secondary, multi‐disciplinary services and trained professionals, high costs to patients, the prevailing obesogenic environment; and stigma experienced by patients within the healthcare services [22].

**FIGURE 1:**
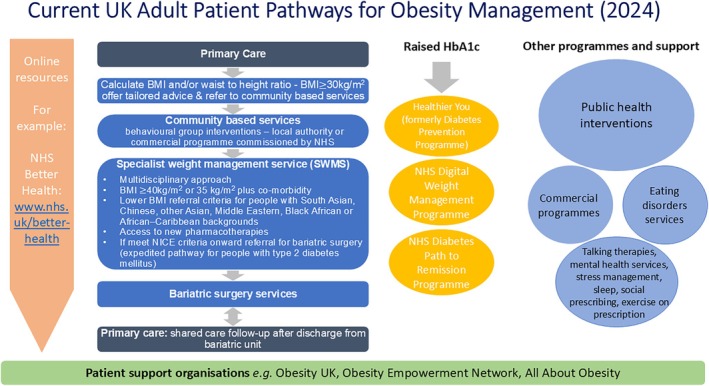
Current UK Adult Patient Pathways for Obesity Management (2024).

In England, there are different criteria for entry to each of the tiered services and each of the national programmes, which means the obesity management pathway/s are very difficult to navigate for both patients and primary care clinicians (see Figure 1 for an overview of current UK adult patient pathways for obesity management). Indeed, the historical lack of joined‐up commissioning and the rigidity of the existing pathway has been criticised and may have delayed onward progress by requiring PLwO to spend excessive time in lower tiered services prior to referral [90]. Hazlehurst et al. argued for a simpler system of services for prevention and treatment, with each person being referred directly to the most appropriate weight management service for their needs [25]. Recent reorganisation of commissioning in England into Integrated Care Systems (ICSs) may also offer an opportunity to develop more flexible pathways. In addition, the more flexible definitions of SWMS given in NICE clinical guidance published this year may help improve access (see Box 2) [19]. The therapeutic options for treating obesity are rapidly evolving, and WMS must continuously review the evidence and guidelines and update their provisions accordingly.

Lack of education of primary care HCPs has been discussed as a potential barrier to accessing services for PLwO, but even when educational interventions have been provided to primary care practitioners there is limited evidence of their efficacy [78, 79]. However, addressing use of language may be of benefit; recent guidance articulates the importance of acknowledging the difficulty on both sides of a weight management conversation and provides practical advice on appropriate language and its potential to reduce stigma [67, 91]. This issue was also highlighted by people living with obesity who provided feedback on the contents of this review (see Box 3).

There is increasing recognition of the complex multifaceted system of determinants underlying obesity and the need for a whole systems approach to obesity (including in the recently updated NICE guidelines) [19]. Therefore, the role of primary care in the prevention and management of obesity, needs to be considered in the wider context. In alignment with this, the World Health Organisation also recommend a ‘Health in all policies’ (HiAP) approach to improving health and reducing inequalities [19, 92]. ‘Real world’ evidence supporting whole system approaches in practice have been demonstrated, such as through the ‘Amsterdam healthy weight approach’ (Netherlands) [93], ‘Shape up Somerville’ (USA) [94] and Romp & Chomp (Australia) [95].

BOX 3 **Some perspectives on this review from people living with obesity.**

We disseminated the final draft of the review paper to two people living with obesity who then discussed their thoughts with authors, H.M.P. and S.E.E. They raised several points:

- The initial contact with the HCP is vital—it takes a lot of courage to present, very important for an HCP to have the right approach to the consultation
- HCP may not be well informed; this can be understandable, but they should at least know where to look for information
- Language and terminology are still not being used correctly—adding to stigma
- Complex referral pathways—PLwO described confusion as to what would be the next step if they presented to primary care
- Moving landscape of management—adding to confusion for all
- A feeling of jumping through hoops to get to definitive treatment such as surgery or medication
- Hopelessness—waiting lists so long it feels the choice is put up with your situation or seek private help
- Potential for new GLP‐1 medications to be very useful—but these are clearly accessible privately in an unregulated manner which is concerning

In 2018, the European Association for the Study of Obesity (EASO) guidance for the management of patients post‐bariatric surgery was published, which highlighted the need for long‐term follow‐up, most likely being delivered within primary care, echoing the NICE guidance for annual reviews in a shared care model [96]. However, there is no specific healthcare funding or established services available to support GPs in England to undertake long‐term care annual reviews, with evidence suggesting that reviews are not being conducted [97]. Given the recent increase in patients having private surgery outside the United Kingdom [98], post‐bariatric surgery care is becoming an increasing issue in the United Kingdom, placing strain on specialist services and primary care. To help GPs manage post‐bariatric surgery patients when no specialist support is available, the British Obesity and Metabolic Surgery Society (BOMSS) launched a GP Hub on their webpages in 2023 (https://bomss.org/gp‐hub/). Similar issues appear to be arising with some PLwO privately procuring new anti‐obesity medications. The place of these highly effective pharmacotherapies has yet to be fully defined, and models of care to support people on the new pharmacotherapies within the NHS are yet to be developed, although a national pilot of new pharmacotherapies for obesity delivered in primary care was proposed [49]. As discussed above, it is likely that primary care will play a key role in this care. The increasing concerns about the impact of private obesity treatments on primary care have brought attention to issues around health inequalities as well as the role of social media in advertising weight management treatments, the lack of regulation and potential risks to patient safety. Consideration of how commercial obesity services could safely be included in a whole systems approach is needed.

## 4 Conclusion

This review has highlighted the role of primary care in obesity management using England as an exemplar. While the reach of primary care offers an unrivalled opportunity to engage with people about prevention and treatment of obesity, to date multiple factors have meant that this opportunity has largely gone unrealised. Relevant factors include low prioritisation by clinicians, workload pressures, geographical variation in services, poor availability of specialist training about obesity, lack of funding and weight stigma. The rapid changes in management with the advent of new pharmacotherapies will be challenging for non‐specialist clinicians to keep abreast of, both in terms of knowledge of the medications themselves and of how they fit into the necessary whole system approach. These factors must be satisfactorily addressed for primary care to play a pivotal role in facilitating access to appropriate treatments, with the new pharmacotherapies offering a significant opportunity for primary care to support patients in accessing the most suitable options.

## Author Contributions

**H.M.P.:** conceptualisation, writing – original draft preparation, writing – review and editing. **K.D.C.:** conceptualisation, writing – original draft preparation, writing – review and editing. **K.C.:** conceptualisation, writing – review and editing. **R.M.:** conceptualisation, writing – original draft preparation, writing – review and editing. **K.W.:** writing – original draft preparation, writing – review and editing. **R.W.:** writing – original draft preparation, writing‐eview and editing. **C.A.H.:** conceptualisation, writing – original draft preparation, writing – review and editing. **S.E.E.:** writing – original draft preparation, writing – review and editing.

## Conflicts of Interest

H.M.P. is a council member for the British Obesity and Metabolic Surgery Society, a member of the NICE quality standards committee for obesity, and an advisory panel member for the UK Coalition for People Living with Obesity. She was a member of the NICE obesity management guidelines(NG246) and an expert advisor to NICE early value assessment of digital technologies for delivering multi‐disciplinary weight management services. She was a member of the steering group for the Obesity Empowerment Network (ended 2024). She has received honoraria for educational events for HCPs from Johnson & Johnson, honoraria for participating in the development and dissemination of an algorithm for the management of adult obesity in primary care supported by arm's length sponsorship from Novo Nordisk, and from Boston Scientific for consultancy work. K.C. is Executive Director for the UK Coalition for People Living with Obesity. He was a director of bariatric surgery, Obesity UK and chair of the European Coalition for People Living with Obesity and a trustee for the Association for the Study of Obesity. He has been a member of the IHCOM board, a Novo Nordisk Disease Experience Expert Panel since 2017, a Patient Advisory Board member for Boehringer Ingelheim since 2020, and Advisory Board member for Roche, and Astra Zeneca (honoraria received for these last four roles). He has received consulting fees from Eli Lilly, Boston Scientific, Liva Health and Zealand. He has received fees for personal lectures from Apollo Endo Surgery, Novo Nordisk SW Europe and J&J Ethicon. C.A.H. has received honoraria for educational events from NovoNordisk and Ethicon.

## Data Availability

Data sharing is not applicable to this article as no new data were created or analyzed in this study.
